# Clinical Outcomes Following SARS-CoV-2 Infection in Pediatric Cystic Fibrosis Patients

**DOI:** 10.7759/cureus.62821

**Published:** 2024-06-21

**Authors:** Andy P Huang, Andrea Espina Rey, Christie G Cherian, Floyd R Livingston

**Affiliations:** 1 Medicine, University of Central Florida College of Medicine, Orlando, USA; 2 Statistics, University of Central Florida College of Medicine, Orlando, USA; 3 Pulmonology, Nemours Children's Hospital, Orlando, USA

**Keywords:** pediatrics, covid-19 outcomes, pulmonary outcomes, sars-cov-2, pediatric pulmonology, covid-19, cystic fibrosis (cf)

## Abstract

Background

Cystic fibrosis (CF) is a genetic disorder of the cystic fibrosis transmembrane conductance regulator chloride channel that leads to impaired mucus clearance in the airways, which leads to deteriorations in lung function and chronic respiratory infection. These effects of CF contribute to the hypothesis that patients with CF may be at increased risk of complications when they catch coronavirus disease 2019 (COVID-19), which swept the world in a global pandemic starting in 2019. Overall, however, the role of CF in COVID-19 has not been well studied, particularly in pediatric patients.

Methods

In this retrospective review, pediatric patients with CF who contracted COVID-19 (3/1/2020-3/1/2023) (N=69) were compared to two equally sized control cohorts of patients with only CF or COVID-19 matched based on demographics and clinical baselines. Occurrences of adverse outcomes (emergency room visits, hospitalizations, CF pulmonary exacerbations, etc.) were assessed for each subject. The mean percentage of predicted forced expiratory volume in 1 second (FEV1%pred) was also assessed for CF patients. Fisher’s exact test assessed differences between the proportions of subjects who experienced each outcome. Independent two-variable *t*-testing assessed mean FEV1%pred differences. Analysis was conducted using IBM SPSS Statistics for Windows, Version 29 (Released 2023; IBM Corp., Armonk, New York, United States) with a significance α=0.05. *Ad hoc* power analysis was conducted using G*Power* *v3.1.

Results

Overall, CF/COVID subjects fared similarly to control groups without either CF or COVID-19 history, including among subgroups stratified based on baseline respiratory function, *P. aeruginosa *colonization status, and COVID-19 vaccination status. One notable finding was that CF/COVID subjects experienced significantly fewer pulmonary exacerbations compared to CF-only subjects (*p*=0.004).

Conclusion

In conclusion, pediatric CF patients performed similarly to their peers without CF with regard to COVID-19 and generally did not demonstrate significant deteriorations in pulmonary function following infection. Lower incidence of pulmonary exacerbations in CF/COVID subjects could be explained by stringent monitoring by parents, quarantine, or close pulmonology follow-up. These findings will provide guidance on management and care for pediatric CF patients with COVID-19.

## Introduction

Cystic fibrosis (CF) is a genetic disorder inherited in an autosomal recessive fashion and primarily involves loss-of-function mutations in the *CFTR *gene, which encodes for CF transmembrane conduction regulator (CFTR), a cAMP-regulated chloride transporter protein [1,2]. CF demonstrates allelic heterogeneity with more than 2,000 known variants, with the most common variant worldwide being the *ΔF508 *mutation, involving the deletion of a phenylalanine residue at position 508 in the CFTR protein [3,4]. CFTR expression in human bronchial tissue is primarily localized to submucosal glandular cells, with defects leading to the abundant production of thick mucus in the bronchi and corresponding small airway obstruction, impaired clearance, inflammation, bronchiectasis, and long-term lung function deterioration [1,4-6]. Aberrant mucus production also facilitates colonization of the airways by various bacterial pathogens like *P. aeruginosa* and *S. aureus*, which leads to chronic infection [1,4]. These manifestations thus necessitate aggressive airway clearance therapy and infection prevention by those with CF in order to prevent long-term deterioration of respiratory function [6].

Ductal systems of other organ systems are also affected by CFTR loss-of-function, manifesting with symptoms such as pancreatic insufficiency, intestinal obstruction at birth, and the obstruction or absence of the vas deferens in men, making CF a pleiotropic disease [1]. However, the most common complications of CF pertain to its pulmonary manifestations, specifically in the form of pulmonary exacerbations (PEx) involving reduced pulmonary function (measured as forced expiratory volume in 1 second, FEV1) and worsening respiratory symptoms such as coughing, wheezing, and mucus production [6]. These exacerbations have been thought to be associated with pulmonary inflammation mediated by elevated levels of pro-inflammatory cytokines like IL-8 and TNFα, reductions in anti-inflammatory cytokines, and elevated toll-like receptor activation [1]. Pulmonary complications, both acute and chronic, are typically managed using airway clearance therapy, antibiotic therapy, anti-inflammatories, and CFTR modulators that act directly on CFTR depending on the patient’s disease genotype [6].

Severe acute respiratory syndrome coronavirus 2 (SARS-CoV-2) is the etiological agent for coronavirus disease 2019 (COVID-19), which spread rapidly across the globe in 2020 in a global pandemic and has led to over 670 million cases and six million deaths as of January 2023 [7,8]. SARS-CoV-2 targets human cells through interactions between the viral spike (S) protein with human angiotensin-converting enzyme 2 (ACE2), which are primarily expressed in lung pneumocytes, goblet cells of the nasal mucosa, and gut enterocytes [7,9]. This tropism corresponds with a clinical manifestation that primarily involves the respiratory system, with signs and symptoms such as cough, dyspnea, hypoxia, pneumonia, and eventually acute respiratory distress syndrome (ARDS) in cases of severe disease [10,11]. The effects of COVID-19 on the respiratory system have led to CF being classified as a risk factor for COVID-19 in guidelines for clinical care and risk stratification in several jurisdictions during the early stages of the pandemic [12].

Previous studies have demonstrated that CF patients are more susceptible to infection by respiratory viruses such as human parainfluenza virus 3 (HPIV3) due to deficiencies in innate immune mechanisms in airway epithelial cells, including reduced activation of nitric oxide synthase 2 (NOS2) and 2’, 5’ oligoadenylate synthase 1 (OAS1) responses to viral infection [13]. Additionally, CF patients have also been shown to experience respiratory complications with respiratory viral infection. In a previous longitudinal cohort study by Armstrong et al., over 50% of infants with CF who were hospitalized for respiratory illness had a respiratory viral infection, with respiratory syncytial virus being the most common virus identified [14]. Other viruses such as influenza and rhinovirus have also been found to induce deleterious effects on pulmonary function in CF patients and increase the duration of antibiotic therapy required for pulmonary exacerbations [15]. Notably, first-time isolation of respiratory pathogens like *P. aeruginosa* and *H. influenzae* was found to be often preceded by symptomatic viral respiratory illness in CF patients [16]. These findings would suggest that CF patients would also be at a greater risk for acquiring COVID-19 and associated complications. Correspondingly, it has been found that CF patients could be at risk for poor clinical outcomes following SARS-CoV-2 infection [17].

Some have suggested that specific CF-related risk factors such as the mean percentage of predicted forced expiratory volume in 1 second (FEV1%pred) < 70% (predicted values standardized from NHANES III [18]) are associated with worse outcomes [19,20]. However, there is also data to suggest that CF patients may actually fare better than non-CF counterparts with regard to COVID-19 infection due to a variety of mechanisms. One study by Bezzerri et al. demonstrated that ACE2 expression and localization are positively correlated with those of CFTR, with the *ΔF508* mutation in particular leading to maldistribution of ACE2 into the endoplasmic reticulum and a corresponding reduction of SARS-CoV-2 replication in vitro [21]. Another study by Abraham et al. hypothesized that elevated systemic ATP pools in CF patients help with survival following COVID-19 infection [22]. Correspondingly, in pediatric CF patients without pre-existing severe lung disease, it has been observed in one international study by Bain et al. that SARS-CoV-2 infection is associated with mild illness no different from those of pediatric patients without CF [23]. However, cases involving pediatric CF patients with a worsening CF trajectory later experiencing pulmonary exacerbations post-COVID diagnosis have been noted [23]. It is unclear, however, if these cases represent a pattern or an exception.

Due to the presence of conflicting evidence on the impact of COVID-19 infection on those with CF, both on COVID-19 prognosis and CF prognosis, we believe there is a need for further study in this area. Studies on the impact specifically on children with CF are even more limited, further highlighting a need for more information, as this would have an impact on guidelines for risk stratification and disease prevention for this cohort. Through this study, we hope to be able to gain a better understanding on how pediatric CF patients are impacted by COVID-19, and how this may implicate risk prevention strategies in this cohort.

Based on current understanding, we hypothesize that pediatric patients with well-managed CF and compensated FEV1%pred will generally exhibit a similar COVID-19 prognosis compared with pediatric patients who do not have CF. However, we also predict that cases involving pediatric CF patients with poorly managed CF, such as those with a low FEV1%pred, would lead to worsened COVID-19 outcomes due to deterioration in respiratory function.

## Materials and methods

Institutional review board approval was obtained from the Nemours Office of Human Subjects Protection (#2008285). For this retrospective review, a cohort of pediatric patients with CF who were diagnosed with COVID-19 between 3/1/2020 and 3/1/2023 was compared to two control cohorts of pediatric patients with either no history of COVID-19 or CF matched based on confounding factors. The overall study methodology is summarized in Table 1. 

**Table 1:** Summary of the study methodology. CF: Cystic fibrosis; BMI: body mass index; FEV1%pred: forced expiratory volume in 1-second predicted; PEx: pulmonary exacerbation; MIS-C: multisystem inflammatory syndrome in children.

Stage	Process
Subject identification	Categorized subjects into the following groups: (1) History of CF and COVID-19 (CF/COVID), (2) History of CF but not COVID-19 (CF), (3) History of COVID-19 but not CF (COVID). First COVID-19 infections were between 3/1/2020 and 3/1/2023, and all CF subjects were diagnosed by this timeframe.
Matching	Matched CF/COVID subjects with CF-only and COVID-only subjects based on age, sex, race, BMI (~March 2020), COVID-19 vaccination status (COVID only), *P. aeruginosa* chronic colonization (CF only, represented by chronic azithromycin therapy), and baseline FEV1%pred (CF only). Independent two-variable t-testing was used to ensure mean age, BMI, and baseline FEV1%pred were not significantly different between groups.
Data collection	(1) CF subjects – determined the mean FEV1%pred, along with occurrence of pulmonary-related ER visits, hospitalizations, ICU admissions, oxygen & ventilator usage, & pulmonary exacerbations (PEx). (2) COVID subjects – determined the occurrence of ER visits, hospitalizations, ICU admissions, oxygen & ventilator usage, COVID antiviral usage & multisystem inflammatory syndrome in children (MIS-C). (3) CF/COVID subjects had variables for both groups collected. All occurrences were recorded only post-COVID infection.
Statistical analysis	Statistical analysis was conducted using IBM SPSS v.29. Independent two-variable t-testing was used to test for significance in difference between FEV1%pred. χ^2^-testing (Fisher’s exact) was used to test for significant differences between proportions of subjects who experienced each outcome. Significance was set at α = 0.05 for all analyses. *Ad hoc* power analysis was conducted using G*Power v.3.1.

Data sourcing

Data was collected from the electronic medical record system of the Nemours Children’s Health system in Florida and Delaware. Sixty-nine patients with confirmed diagnosis of CF and COVID-19 within the timeframe 3/1/2020 to 3/1/2023 based on medical records were identified for the study (CF/COVID) group. Patients with CF who have no history of COVID-19 (CF group) and patients with a history of COVID-19 with no diagnosis of CF (COVID group) were also identified for control matching.

Matching

For all subjects, the following variables will be extracted for purposes of matching: age, sex, race, and body mass index (BMI). CF/COVID patients were matched with one subject from both CF and COVID controls groups based on these factors. Additionally, COVID-19 vaccination status was used for matching with COVID controls. Chronic azithromycin therapy, which is representative of chronic *P. aeruginosa* colonization, was only used for matching with CF controls. Mean baseline FEV1%pred obtained from the interval 3/1/2020-3/1/2023 was also obtained and used for matching the CF control group. For CF/COVID subjects, mean baseline FEV1%pred was calculated using measurements between 3/1/2020 and the date of first COVID-19 infection. Subjects who do not have FEV1%pred records, either due to incomplete medical records or young age, were not matched using this metric. Independent two-variable t-testing was used to ensure that no significant differences in mean age, BMI, and baseline FEV1%pred existed between final groups (Table 2).

**Table 2:** Matched group demographics. CF/COVID group subjects are matched with CF-only and COVID-only control subjects based on the displayed factors. Chronic azithromycin therapy is representative of *P. aeruginosa* chronic colonization status. Significance of differences for age, BMI, and baseline FEV1%pred determined using independent sample t-testing (α = 0.05). CF: Cystic fibrosis; BMI: body mass index; FEV1%pred: forced expiratory volume in 1-second predicted.

	CF/COVID	CF	COVID	p-value
Total	69	69	69	
Male	35 (50.7%)	35 (50.7%)	35 (50.7%)	
Female	34 (49.3%)	34 (49.3%)	34 (49.3%)	
White/Caucasian	58 (84.1%)	58 (84.1%)	58 (84.1%)	
Black/African American	1 (1.4%)	1 (1.4%)	1 (1.4%)	
Hispanic/Latino	8 (11.6%)	8 (11.6%)	8 (11.6%)	
Asian	0 (0.0%)	0 (0.0%)	0 (0.0%)	
Other races	2 (2.9%)	2 (2.9%)	2 (2.9%)	
COVID-19 vaccination	29 (42%)	-	29 (42%)	
Chronic azithromycin	5 (7.2%)	5 (7.2%)	-	
Mean age	7.30 (SD = 5.424)	7.06 (SD = 5.000)	7.22 (SD = 5.139)	0.782, 0.923
Mean BMI	17.49 (SD = 2.123)	16.92 (SD = 1.935)	17.80 (SD = 2.712)	0.102, 0.457
Mean baseline FEV1pred%	97.86% (N = 39, SD = 17.03%)	94.52% (N = 39, SD = 13.31%)	-	0.339

Data collection

To assess clinical outcomes, the following metrics were extracted: occurrences of pulmonary-related emergency room (ER) admissions, hospitalizations, intensive care unit (ICU) admissions, oxygen therapy usage, and ventilator usage. For the CF/COVID-CF comparison, the mean post-COVID FEV1%pred and the occurrences of CF-related pulmonary exacerbations were also recorded. For the CF/COVID-COVID comparison, the occurrences of multisystem inflammatory syndrome in children (MIS-C) and instances of SARS-CoV-2 antiviral usage were also recorded. Occurrences are counted if they occur between the date of COVID-19 infection and 3/1/2023 for subjects who are, and between 3/1/2020 and 3/1/2023 for subjects not infected. All data is collected with and managed using REDCap electronic data capture tools hosted by Nemours Children’s Health [24,25].

Statistical analysis

χ^2^-testing (Fisher’s exact tests) were used to test for significant differences between the proportions of patients who experienced each outcome between groups. Independent sample two-variable t-testing was used to test for significant differences in mean FEV1%pred. The significance level (α) is set at 0.05. Statistical analysis was conducted using IBM SPSS Statistics for Windows, Version 29 (Released 2023; IBM Corp., Armonk, New York, United States). Post hoc power analysis was conducted using G*Power v.3.1 [26].

## Results

**Figure 1:**
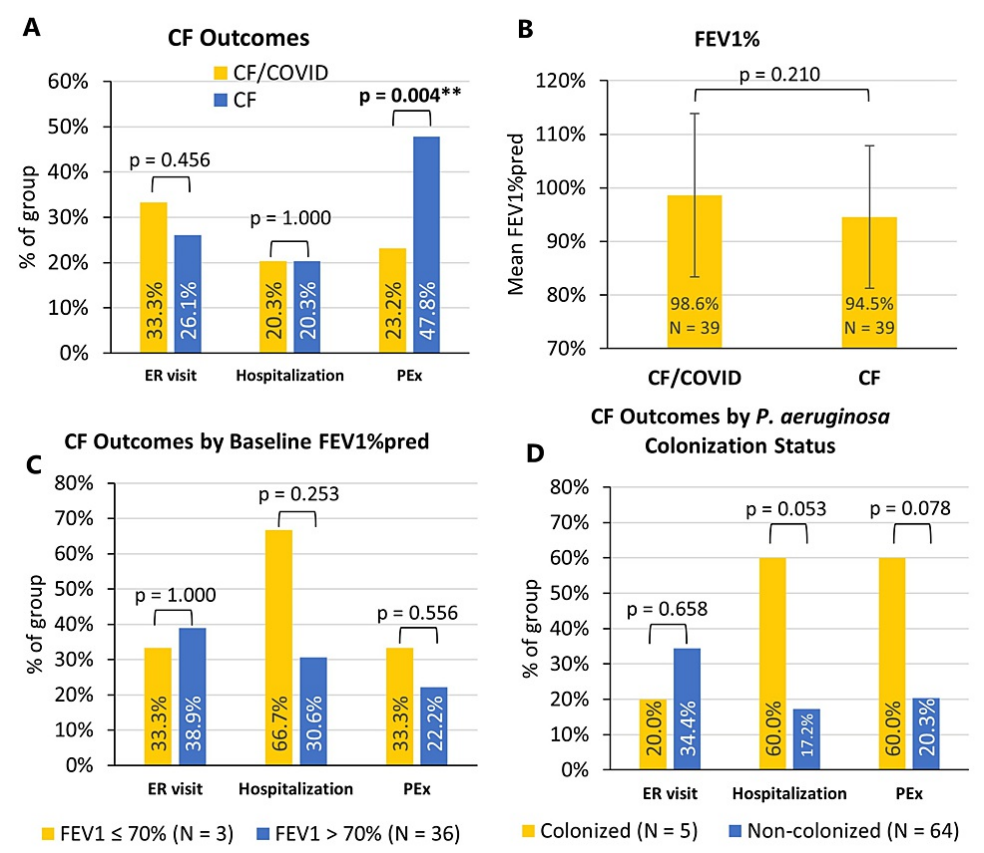
CF outcomes. (A) Occurrences of CF-associated outcomes in CF patients with and without COVID-19 history. Occurrences of ICU admissions (N = 1), oxygen usage (N = 2), and ventilator usage (N = 0) were insignificant and were not further analyzed. Significance was determined using χ^2^ testing (Fisher’s exact) (α = 0.05). (B) Mean FEV1%pred for CF patients with and without COVID-19 history. Significance was determined using independent sample t-testing (α = 0.05). (C) CF outcomes for CF/COVID subjects stratified based on baseline FEV1%pred. (D) CF outcomes for CF/COVID subjects stratified based on *P. aeruginosa* colonization status. ** Statistical power >80% as determined via *post hoc* analysis. CF: Cystic fibrosis; FEV1%pred: forced expiratory volume in 1-second predicted; PEx: pulmonary exacerbation.

When comparing outcomes of the CF/COVID group with the CF group, overall differences in the rate of pulmonary-related ER admission, hospitalization, and post-infection FEV1%pred were insignificant (p > 0.05) (Figures 1A, 1B). However, the rate of PEx was significantly higher in the CF group compared to the CF/COVID group (p = 0.004) (Figure 1A). When the CF/COVID group was stratified based on baseline FEV1%pred, differences in the rate of pulmonary-related ER admission, hospitalization, and PEx were insignificant (Figure 1C). When the CF/COVID group was stratified based on *P. aeruginosa* colonization status, differences in the rate of pulmonary-related ER admission, hospitalization, and PEx were insignificant (Figure 1D). Occurrences of severe outcomes like ICU admission, oxygen and ventilator usage, SARS-CoV-2 antiviral usage, and MIS-C were uncommon across all study groups. Only three subjects across the entire study required ICU admission, two required oxygen therapy, three required monoclonal antibody treatment for COVID-19, one experienced MIS-C, and none required mechanical ventilation.

**Figure 2:**
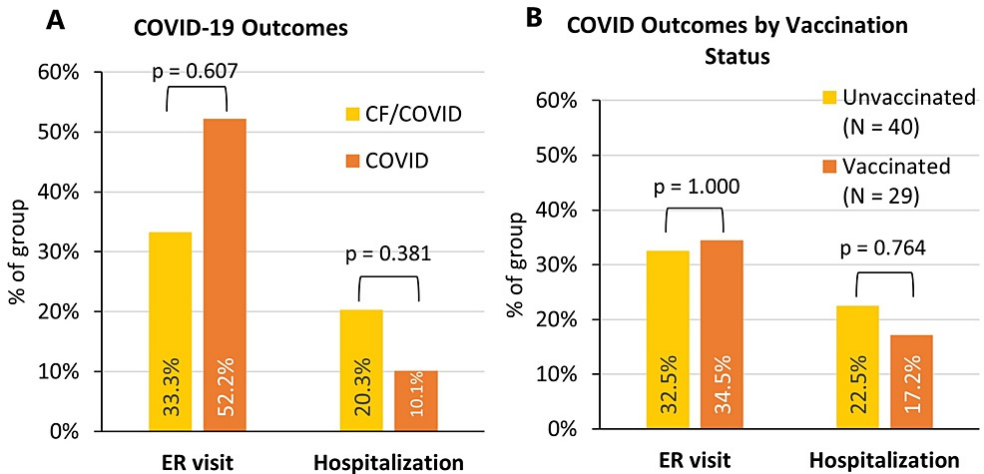
COVID-19 outcomes. (A) Occurrences of COVID-associated outcomes in patients with and without CF. Occurrences of ICU admissions (N = 3), oxygen usage (N = 2), ventilator usage (N = 0), antiviral usage (N = 3), and MIS-C (N = 1) were insignificant and were not further analyzed. Significance was determined using χ^2^ testing (Fisher’s exact) (α = 0.05). (B) COVID-19 outcomes for CF/COVID subjects stratified based on COVID-19 vaccination status. CF: Cystic fibrosis.

When comparing outcomes of the CF/COVID group with the COVID group, overall differences in the rate of pulmonary-related ER admission and hospitalization were insignificant (p > 0.05) (Figure 2A). When the CF/COVID group was stratified based on COVID-19 vaccination status, differences in the rate of pulmonary-related ER admission and hospitalization remained insignificant (Figure 2B).

## Discussion

Overall, pediatric patients with CF fared well against both COVID-19 and CF when they contracted COVID-19. This would corroborate observations like those by Bain et al. who have noted that pediatric CF patients generally experience mild symptoms with COVID-19 and perform well [23]. Paradoxically, CF/COVID patients exhibited a significant decrease in PEx occurrence (Figure 1A). We believe this could be explained by more stringent monitoring of infection and symptoms by parents, less exposure to pathogens from the environment due to quarantine, or regular follow-up with pulmonology when CF patients contract COVID-19. However, this may also lend support to the theory that reduced expression and maldistribution of ACE2 in CF patients may be beneficial with regard to COVID-19 prognosis [21]. These findings are generally reassuring, although the relatively small sample size used does present limitations in the generalizability of our findings. Additionally, the overall small incidence of severe outcomes like ICU admission, oxygen and ventilator usage, SARS-CoV-2 antiviral usage, and MIS-C across our sample suggests that pediatric patients in general rarely progress to severe outcomes or require advanced interventions in the context of COVID-19 infection and further corroborates our findings.

Baseline pulmonary function and *P. aeruginosa* chronic colonization are both representative of less adequately controlled CF, which prompted us to stratify the CF/COVID group based on these factors to determine if these groups may exhibit worse outcomes. An FEV1%pred of less than 70% in particular is considered by the Cystic Fibrosis Foundation to be the boundary between mild and moderate pulmonary function reduction [27]. FEV1%pred < 70% is also a group that was previously suggested to be associated with worsened outcomes with COVID-19 [19,20]. No significant differences in the CF outcome were noted for both FEV1%pred < 70% and *P. aeruginosa* colonized groups, although the small sample size for both (N = 3 and 5 respectively) limited the power of this analysis. We do note that the FEV1%pred < 70% group had an elevated but not significant hospitalization rate (66.7% vs. 30.6%), while the *P. aeruginosa* chronic colonization group had elevated but not significant rates of hospitalization (60.0% vs. 17.2%) and PEx (60.0% vs. 20.3%). Further studies with larger sample sizes for each cohort and possibly additional cohorts for more severe disease (i.e. FEV1%pred < 40%) should be done to determine if CF patients with reduced pulmonary function or chronic colonization experience worse outcomes than those without these complications.

Regarding outcomes of COVID-19, we noted no significant differences in ER visits or hospitalizations between patients with and without CF, which holds up for both patients vaccinated and not vaccinated against COVID-19. This is again consistent with previous observations of mild COVID-19 courses in this population and further points to an overall reassuring picture regarding CF and COVID-19 in pediatrics.

## Conclusions

With the continued prevalence of COVID-19 in the community, it is important to better understand how the virus affects those with CF, who are already at risk for a variety of infections from the environment and pulmonary function deterioration. Our findings suggest that pediatric CF patients overall fare with COVID-19 similarly to their peers who do not have CF and do not demonstrate significant deteriorations in pulmonary function following infection. Attentive care and close medical follow-up may even contribute to better pulmonary outcomes following COVID-19 infection. However, we do note that subgroups of CF patients with lower baseline pulmonary function or *P. aeruginosa* colonization may fare worse with COVID-19, which cannot be concluded due to limited statistical power in our study. Therefore, further studies should be done to better understand how COVID-19 affects children with CF, and how COVID-19 in this population should be managed.
